# Nondestructive detection and identification of electrically active threading dislocations in n^+^-SiC substrates

**DOI:** 10.1039/d5na00970g

**Published:** 2025-12-03

**Authors:** Irwan Saleh Kurniawan, Russel Cruz Sevilla, Hsiu-Ming Hsu, Ruth Jeane Soebroto, Chii-Bin Wu, Ji-Lin Shen, Hsiu-Ying Huang, Wen-Chung Li, Chi-Tsu Yuan

**Affiliations:** a Department of Physics, Chung Yuan Christian University Taoyuan Taiwan estion53@yahoo.com.tw ctyuan@cycu.edu.tw; b Research Center for Semiconductor Materials and Advanced Optics, Chung Yuan Christian University Taoyuan Taiwan; c LEAP Semiconductor Corporation Taoyuan Taiwan wenchunglee@leap-semi.com

## Abstract

Threading dislocations (TDs) are the most abundant extended defects in highly n-doped SiC (n^+^-SiC) substrates. Notably, only a small subset, those hosting deep states (DS-TDs), can potentially impact device operation. However, selective detection of such electrically active DS-TDs using conventional photoluminescence (PL) techniques remains challenging due to universal PL quenching across all defects. Here, we develop confocal subsurface defect-PL spectro-microscopy to selectively detect screw-component DS-TDs (DS-STDs) in n^+^-SiC substrates. By directly photoionizing the occupied deep states, DS-STD-specific emissions can be activated. Such inherent deep-level emissions of dislocation lines, combined with external surface-state emissions at the etch pits, enable the reconstruction of 3D images with high contrast for the partially etched DS-STDs. This approach overcomes the limitations of conventional PL and paves the way for non-destructive, in-line inspection of electrically active dislocations even in highly doped SiC substrates.

## Abstract 2

Three-dimensional photoluminescence mapping was employed to visualize partially etched dislocations in a SiC substrate, enabling a potential method for identification of killer threading dislocations responsible for device degradation.

## Graphical abstract

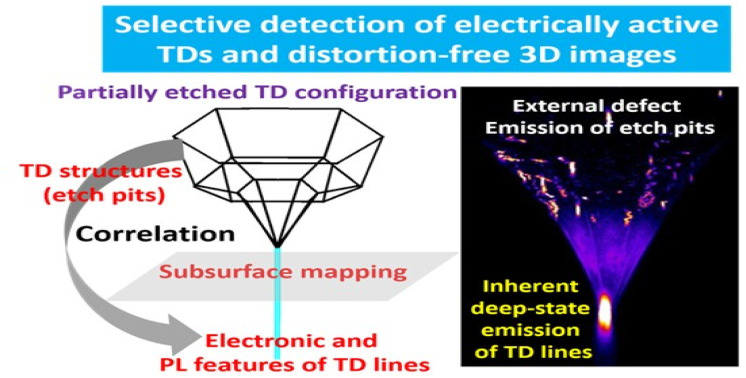

## 1. Introduction

Silicon carbide (SiC) is a wide-bandgap semiconductor with several promising material properties, including a large electrical breakdown field, high thermal conductivity, and high electron saturation velocity.^1^ These properties enable SiC-based power devices to operate under high-temperature and high-voltage conditions, as well as in harsh environments.^2^ Despite significant technological advances in SiC power devices, a high density of threading dislocations (TDs) still exists in commercially available SiC substrates.^3–5^ These TDs can further propagate into the epitaxial layers and terminate on the surface and thus would negatively affect the crystal structures and surface morphology of the epitaxial layers, potentially deteriorating the device performance and reliability.^3,6–10^ In addition, TDs can also act as nucleation sites for the formation of other extended epitaxial defects, such as carrot and triangular defects.^8,11^

Certain TDs have been identified as leakage current paths and charge trapping centers, thus classifying them as device-killing extended defects for power devices.^12–14^ These electrically active TDs host deep states within the bandgap (hereafter referred to as DS-TDs), spatially distributed along the entire dislocation lines. As a result, charge carriers can be trapped, generated, and recombined through these deep states, contributing to reverse leakage current *via* mechanisms such as trap-assisted tunneling and the Poole–Frenkel emission effect.^15–17^

It should be noted that such DS-TDs constitute only a small subset of all TDs but play a critical role in determining device yield, performance, and reliability.^12,18,19^ For instance, X. Zhang *et al.* investigated the correlation between the failed dies of Schottky barrier diodes (SBDs) and threading screw dislocations (TSDs) in the substrates. They found that TSDs were observed around the breakdown sites of most failed dies, but not *vice versa*, as abundant TSDs existed without causing device breakdown.^18^ In addition, A. Severino *et al.* investigated the correlation between TDs and failed MOSFET devices after reliability testing and also emphasized the roles of certain TDs in substrates.^12^ Recently, dislocation-related leakage current paths in 4H-SiC were investigated using a combination of conductive atomic force microscopy and KOH etching.^19^ The study revealed that both TSDs and TMDs have a greater impact on reverse leakage current compared to TEDs.

Due to their critical roles in power devices, the development of optical techniques for nondestructively detecting TDs, particularly electrically active DS-TDs in SiC substrates, is highly desirable.^20–22^ Photoluminescence (PL)-based methods are noncontact optical techniques commonly used to nondestructively inspect various defects in semiconductors based on defect-induced band-edge emission quenching.^23–25^ In general, defects can trap the charge carriers and act as nonradiative recombination centers, thus reducing band-edge emissions and forming a PL-dark region.^26^ Consequently, the intensity difference in band-edge emissions between the background matrix and the defects can create imaging contrast, providing information about defect locations.

So far, several types of extended defects, such as TDs and stacking faults, have been visualized using PL-quenching imaging techniques in SiC epilayers with low dopant concentrations.^26,27^ By laboriously mitigating the Z_1/2_ centers by post-oxidation, etching and passivation, TDs in SiC epilayers can be observed using such PL-dark mode in band-edge PL mapping.^28^ These additional post-treatments are required for reducing the defect density and increasing the carrier lifetimes, thereby enhancing the imaging contrast.^29^ Unfortunately, this technique fails in highly n-doped substrates with high defect/impurity density and short carrier lifetimes, where imaging contrast is diminished due to universal band-edge PL quenching caused by abundant background defects/impurities.^30,31^ More importantly, conventional band-edge PL quenching methods cannot selectively visualize electrically active DS-TDs and provide crucial electronic information about their defect nature.

To address these issues, we developed a confocal subsurface defect-PL spectro-microscopy technique with a PL-active mode, enabling selective detection of electrically active screw-TDs with deep states (DS-STDs) in n^+^-SiC substrates. By photo-ionizing the occupied deep states, only DS-STDs can be brightened among all STDs, thus generating broadband visible-range deep-state emissions. Such DS-STD-specific emissions enable 2D subsurface imaging with ultrahigh contrast, while facilitating the reconstruction of distortion-free 3D images. Additionally, we investigated the correlation between etch pit morphology and optical features of dislocation lines to further identify the defect nature of DS-STDs. Our work demonstrates a powerful optical technique for nondestructively detecting DS-STDs even in highly n-doped SiC substrates, while providing valuable insights into their optical fingerprints and defect characteristics.

## 2. Results

### 2.1. Physical concept for selective detection of DS-TDs

In the conventional band-edge PL-quenching method, the degree of PL-quenching depends on the defect nature and carrier diffusion lengths, thus creating a trade-off between imaging contrast and spatial resolution in indirect-bandgap SiC. When target defects dominate, such as TDs in post-treated n^−^-SiC epilayers, acceptable imaging contrast can be achieved if these defects efficiently quench band-edge PL. However, this comes at the expense of poor spatial resolution due to long carrier diffusion lengths, a limitation referred to as carrier-diffusion-limited spatial resolution.^28^ In contrast, in n^+^-SiC substrates with abundant defects/impurities, universal PL quenching by all background defects/impurities significantly reduces the image contrast of target defects. These limitations pose a significant challenge for the detection of TDs with both high spatial resolution and imaging contrast in highly n-doped SiC substrates. Additionally, such band-edge PL-quenching techniques cannot selectively distinguish electrically active DS-TDs from benign TDs and provide no electronic information about defects.

**Fig. 1:**
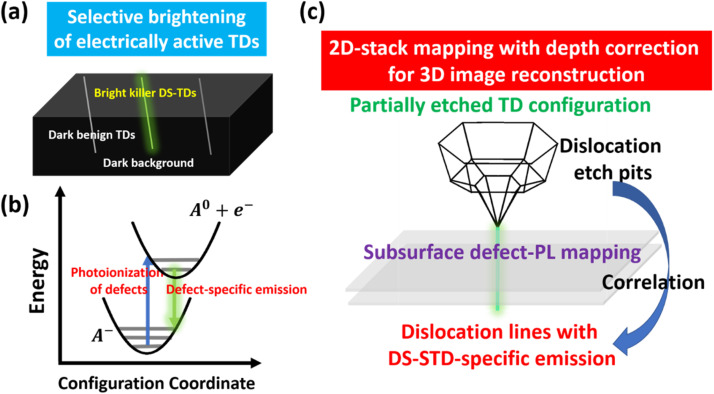
Physical concept for selective detection of DS-TDs: (a) schematics of selective brightening of DS-TDs among all TDs. (b) Direct photoionization and defect emission of acceptor-like deep states based on a configuration coordination diagram. (c) Illustration of the reconstruction of 3D images for partially etched dislocations by 2D-stack subsurface mapping.

Unlike conventional PL-dark mode imaging, we aim to develop an optical technique that exclusively visualizes the DS-TDs among TDs in a PL-active mode, as schematically illustrated in Fig. 1(a). Such electrically active DS-TDs can also become optically active by direct photoionization of their occupied deep states. Fig. 1(b) illustrates the physical concept underlying our approach based on a configuration coordinate diagram for describing direct defect photoionization and defect emission processes, which has been applied for the explanation of electronic–vibronic transitions of deep-level defects in wide-bandgap semiconductors.^32,33^

To elucidate the defect photoionization and following emission processes, we assume that the DS-TDs involve negatively charged acceptor-type deep states, which serves as an illustrative example.^34,35^ By direct photoionization, the defect system can form coordinate-displaced excited states, consisting of free electrons in the conduction band and localized bound-holes at acceptor-type deep states (A^−^ + *hν*_SG_ → A^0^ + e^−^). Subsequently, the free electrons can recombine with the bound holes (A^0^ + e^−^ → A^−^ + *hν*_DPL_), producing defect-specific emission (defect-PL), which would be a specific optical feature for defect identification.

To test this hypothesis, defect-selective etching was intentionally employed to delineate dislocation etch pits (DEPs) in n^+^-SiC substrates, as illustrated in Fig. 1(c). The anisotropic etching process preferentially removes atoms at an enhanced rate in the strained regions surrounding dislocations, thereby making the DEPs visible on the surface while leaving the corresponding dislocation lines beneath.^35^ Revealing DEPs is beneficial for locating dislocations for subsequent optical probing and provides valuable structural indications; however, detailed structural information cannot be determined solely from their DEP morphology. It should be emphasized that the main advance of this work lies in the optical detection and characterization of electrically active TDs with deep states, as well as in establishing their correlation with etch-pit morphology. In fact, pre-etching the samples is not necessary for TD inspection once the optical fingerprints of electrically active DS-TDs are identified, which is the main objective of this study.

With the assistance of the DEPs, we can apply our developed technique, confocal subsurface defect-PL spectro-microscopy, to probe individual TD lines beneath the DEPs (Fig. 1(c)). Probing deep-subsurface TD lines, away from the DEPs, is crucial to capture the inherent deep-state emissions specific to the dislocation lines of DS-TDs. This approach helps eliminate interference from extrinsic surface-state emissions on the hexagonal facets of the DEPs, which can be unavoidably introduced during the chemical etching process.

### 2.2. Selective brightening of individual screw-type DS-TDs

**Fig. 2:**
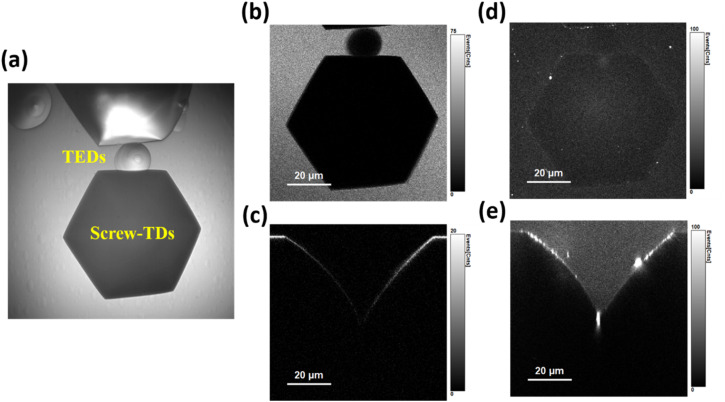
Confocal dual-mode mapping for screw-type TDs: (a) OM images of dislocation etch pits, containing the main target defects, screw-TDs. (b and c) Laser backscattering images of surface and cross-sectional planes for a selected screw-type TD, and (d and e) the corresponding defect-PL images.

Fig. 2(a) presents the optical microscopy (OM) image of revealed DEPs in n^+^-SiC substrates (Fig. S1 for more details). Two types of etch pits with distinct sizes were clearly observed. The morphology of the DEPs, including sizes, shapes, and depth profiles, is influenced by several factors, such as Burgers vectors and dopant concentrations.^36^ As a result, DEPs can provide certain structural information about the underlying TDs. The smaller hexagonal DEPs surrounded by the rounded shells are associated with threading edge dislocations (TEDs). In contrast, larger hexagonal DEPs can be attributed to screw-type TDs (STDs), including both threading screw dislocations (TSDs) and threading mixed dislocations (TMDs). However, a definitive distinction between TSDs and TMDs cannot be made solely based on sizes, owing to inherent size heterogeneity.

Here, we focus primarily on STDs owing to their significant influence on device performance and reliability, serving as leakage current paths and potential nucleation sites for other extended defects within epitaxial layers.^12,18,19^ After selecting specific STDs, confocal dual-mode mapping was conducted, including both laser backscattering mode and defect-PL mode, to simultaneously examine the surface DEPs and subsurface TD lines, as well as perform cross-sectional mapping (without depth correction) for the partially etched TD configuration. Fig. 2(b) and (c) present laser backscattering images of the surface and cross-sectional planes for a selected STD. This direct depth-profile mapping has been used for distinguishing the types of TDs, but it unfortunately suffers from significant imaging distortion due to spherical aberration caused by the large refractive index mismatch between the sample and the environment.^36,37^ Therefore, obtaining calibrated depth profiles is essential for accurate analysis, as will be demonstrated later.

The corresponding defect-PL images are shown in Fig. 2(d) and (e), revealing numerous bright defects on the hexagonal facets of the DEPs, which were externally introduced by the defect-selective etching process. Notably, such foreign defects are absent during nondestructive TD inspection (without chemical etching) and are therefore irrelevant to this study. However, they can brighten the DEP region in the partially etched TD configuration. Remarkably, bright emissions spatially distributed along the TD lines beneath the DEPs are clearly visible in the cross-sectional defect-PL mapping. It should be noted that these emissions originate specifically from the dislocation lines rather than from the etch pits, owing to spatial filtering through the confocal pinhole. Moreover, such unique emissions appear only in a small subset of STDs and can be attributed to those possessing inherent deep states, herein referred to as DS-STD-specific emissions.

### 2.3. 2D subsurface defect-PL mapping of DS-STD-specific emissions

To further investigate DS-STD-specific emissions and evaluate their effectiveness for defect inspection, we conducted 2D subsurface defect-PL mapping of dislocation lines beneath the DEPs (illustrated in Fig. 1(c)). By leveraging deep-subsurface excitation and confocal detection, our technique effectively bypasses interferences from both subsurface damage and etching-induced defects, enabling the collection of DS-STD-specific emissions from the dislocation lines.

**Fig. 3:**
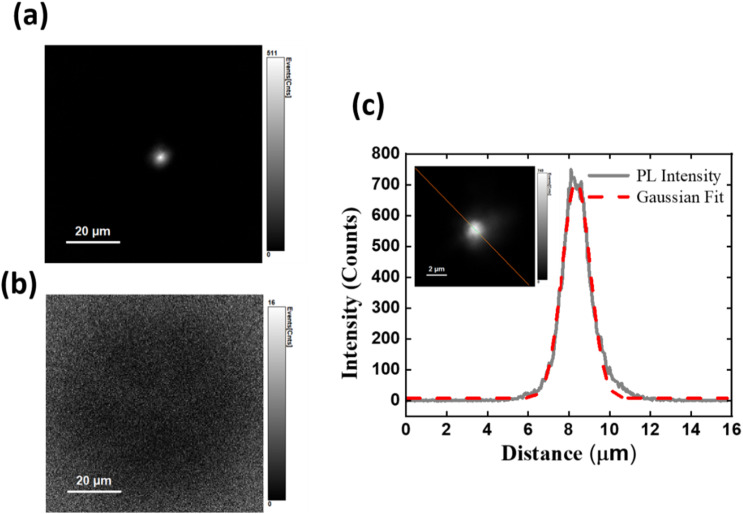
2D subsurface defect-PL mapping for DS-STDs and STDs: (a) subsurface defect-PL image of the DS-STDs, showing a bright emission spot with high imaging contrast and spatial resolution, and (b) the corresponding image of the STDs, which lacks the emission spots. (c) The intensity profile of the emission spots in the subsurface defect-PL mapping with the inset displaying an enlarged view of the defect-PL image.

Fig. 3(a) and (b) present representative 2D subsurface defect-PL images for two STD lines with similar DEP morphologies (additional data available in Fig. S2). In Fig. 3(a), a bright emission spot attributed to DS-STD-specific emissions is clearly visible, exhibiting ultrahigh imaging contrast and spatial resolution even in highly n-doped SiC substrates, referred to as bright DS-STDs. In contrast, Fig. 3(b) shows no such emission, and these are thus classified as dark STDs. Notably, dark STDs can still exhibit bright DEPs due to externally created defect emissions, but appear dark along the dislocation lines due to the absence of inherent deep states. This finding demonstrates that structurally similar STDs (reflected in comparable DEPs) exhibit distinct electronic transitions. The pronounced disparity indicates that the occurrence of deep-state emissions is not solely determined by the TD types. Instead, additional, unidentified factors play a crucial role in synergistically influencing the formation of deep electronic states.

To quantify the imaging contrast, we used the contrast ratio metric, defined as 
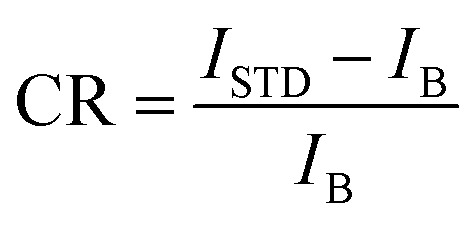
, where *I*_STD_ and *I*_B_ denote the mean of defect-PL intensity of DS-STD-specific emissions and the background intensity, respectively. A larger CR value indicates better imaging contrast and higher visibility for defect inspection. Fig. 3(c) presents the intensity profile of the bright spots along the dotted lines (inset in Fig. 3(c)). The full width at half maximum (FWHM) of the intensity profile is ∼1.3 µm, and an ultrahigh CR value of ∼700 was achieved. This performance is particularly challenging for TD inspection based on conventional nondestructive techniques, such as band-edge PL quenching and X-ray topography.^20,28,38^

It is observed that the spot sizes of DS-STD-specific emissions in defect-PL imaging, even when probing deep subsurface regions in SiC substrates with high refractive index mismatch, are approximately ∼1.3 µm, primarily constrained by optical diffraction. In contrast, spatial resolution in conventional PL quenching and X-ray topography is often largely degraded by the convolution of carrier diffusion lengths or strain fields. The underlying detection mechanism of our technique, PL-active mode *via* deep-state emission, ensures immunity to these limitations. Therefore, we refer to this as optical diffraction-limited spatial resolution, in contrast to carrier diffusion-limited spatial resolution. Consequently, our approach is applicable across all regions of SiC materials and devices. As a result, our unique technique can be applied for probing individual DS-TDs within defect clusters, which may critically impact device performance.^18^

### 2.4. 3D image reconstruction from depth-corrected 2D-stack mapping

By combining the DS-STD-specific emissions from dislocation lines with the etching-induced defect emissions from the DEPs, a comprehensive 3D image of the entire DS-STDs can be obtained. These 3D images of the partially etched TDs can provide valuable insights into the correlation between dislocation structures (revealed by DEPs) and optical/electronic characteristics (derived from the specific emissions of dislocation lines). To this end, 2D-stack defect-PL mapping was performed from the surface into the deep subsurface (∼110 µm) for both bright DS-STDs and dark STDs. Due to spherical aberration caused by the refractive-index mismatch between the immersion medium and the samples, focal depth correction was applied to ensure further reconstruction of 3D images (SI S3 for more details).^37^ This axial correction minimizes imaging distortion, allowing for more precise analysis of DS-STDs.

**Fig. 4:**
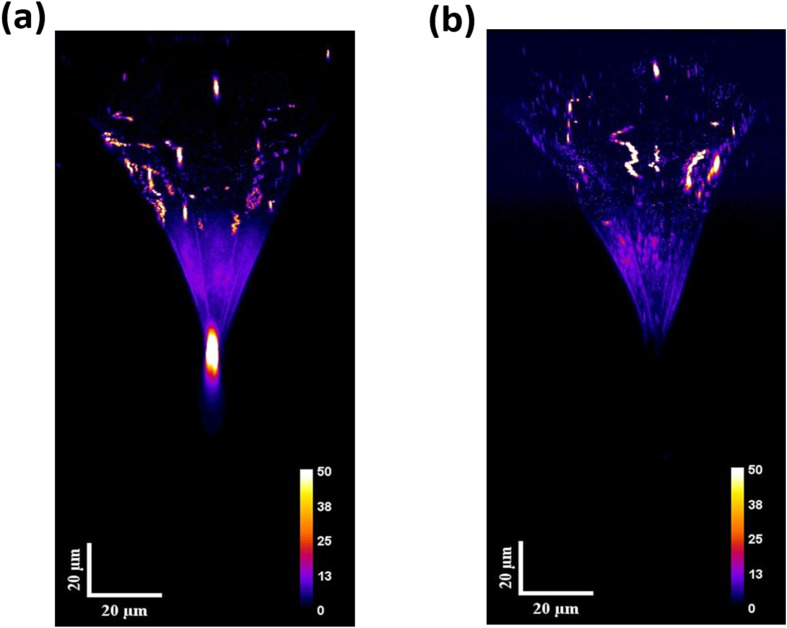
3D image reconstruction from 2D-stack mapping with depth correction: (a) 3D image of entire bright DS-STDs, revealing both DEPs and dislocation lines. (b) 3D images of dark STDs, showing only DEPs, with no emission from dislocation lines.

**Fig. 5:**
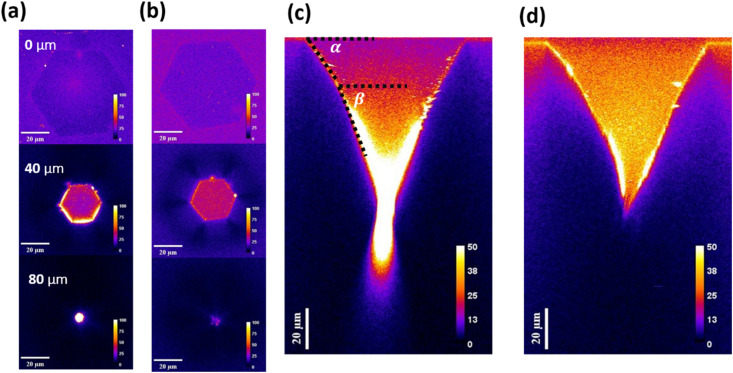
Lateral and cross-sectional views from different angular slices of 3D images: (a and b) lateral slicing of the constructed 3D images, showing both bright DS-STDs and dark STDs. (c and d) Cross-sectional slicing of the constructed 3D images, providing insights into the structural and optical characteristics of DS-STDs and STDs.

Fig. 4(a) and (b) show the 3D images of bright DS-STDs and dark STDs, reconstructed from 2D-stack mapping with depth correction. For bright DS-STDs, both DEPs and tilted dislocation lines are clearly visible, whereas only the DEPs are observed for dark STDs. To investigate potential correlations between the DEP morphology (related to TD structures) and DS-STD-specific emissions, lateral-slicing images at various depths (0, 40, 80 µm) were extracted from 3D images for DS-STDs and STDs, as shown in Fig. 5(a) and (b). Additionally, cross-sectional images were also generated by orthogonal slicing, as depicted in Fig. 5(c) and (d). Using our technique, we can reconstruct depth-corrected 3D images and extract 2D images of the partially etched TD configuration for two structurally similar but electronically distinct STDs, providing deeper insights into electrically active DS-STDs. It should be noted that our optical technique cannot unequivocally distinguish TSDs from TMDs based solely on etch-pit morphology and inclination angles, but it does provide optical access to structural information for both defect types.

By carefully examining lateral and cross-sectional images, we found that the DEPs of both STDs exhibited similar combined features, that is, an end tip beneath the hexagonal stage, which is the characteristic of the TMDs.^39,40^ In this case, two inclination angles relative to the surface plane, denoted as *α* and *β*, were defined for the analysis of depth profiles (Fig. 5(c)). A detailed comparison of the depth profiles revealed that bright DS-STDs typically exhibited a larger difference between *α* and *β* (here *β* − *α* ∼ 15°).

We have applied our technique to probe over 300 individual STDs, aiming to gather statistical data and quantify the prevalence of electrically active DS-STDs among STDs. Interestingly, the majority of STDs (∼93%) exhibited no deep-state emissions from their dislocation lines, indicating the absence of deep states for most STDs. It should be noted that this prevalence is specific to the substrate type, doping concentration, and measurement conditions and should not be generalized as a universal value. This finding is consistent with the previous reports based on electrical measurements in SiC power devices, which also suggested that only a small subset of STDs adversely impacts device performance and reliability.^12,18,19,41^

### 2.5. Defect-PL spectra of deep-state emissions

**Fig. 6:**
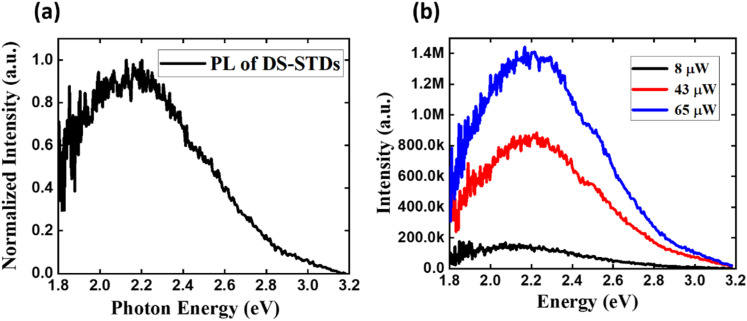
Defect-PL spectra of deep-state emissions in bright DS-STDs. (a) Normalized defect-PL spectra of DS-STDs, exhibiting a peak maximum at 2.17 eV. (b) Power-dependent defect-PL spectra, revealing a blue shift in peak positions with increasing excitation power.

Our defect-PL-active mode can also capture the crucial electronic characteristics of DS-STDs. Consequently, we measured the defect-PL spectra of DS-STD-specific emissions to gain electronic insights into the deep states, as shown in Fig. 6(a). The spectrum exhibits a broad line shape spanning the entire visible range, with a maximum at ∼2.17 eV. This broadband emission indicates the presence of multiple deep states within the bandgap, including both donor-type and acceptor-type states (Fig. S4 for more details). Such broadband, visible-range deep-state emissions, spatially aligned along the dislocation lines and energetically distributed within the bandgap, are unique to DS-STDs. Such deep states enable DS-STDs to spatially bridge the terminals, providing continuous energy levels that form leakage current paths through defect-assisted tunneling or Poole–Frenkel emission mechanisms. As a result, DS-STD-specific emissions can serve as distinctive optical fingerprints for identifying device-relevant TDs *via* nondestructive optical methods in the visible range.

To further investigate their electronic nature, power-dependent deep-state emission measurements were performed, as shown in Fig. 6(b), revealing a slight blue shift in the defect-PL maximum with increasing excitation power, suggesting donor–acceptor-pair (DAP)-type emissions. Notably, these DAP-type emissions are attributed to donor-type and acceptor-type defect states created within the bandgap, rather than impurity-related donors and acceptors, as will be further discussed later.

## 3. Discussion

### 3.1. Comparison of PL-based techniques for TD inspection

T. Kimoto *et al.* demonstrated a PL-based method using µ-PL mapping of band-edge emissions at 390 nm to visualize the TEDs and TSDs in SiC epilayers with low dopant concentrations.^26,28^ However, pre-treatment processes such as oxidation, etching, and passivation are required before PL measurement. These treatments mitigate the defects and extend carrier lifetimes, thereby improving the visibility of dislocations.^29^ By correlating µ-PL intensity imaging with the DEPs, they established a one-to-one correspondence, enabling the differentiation of TEDs and TSDs based on varying degrees of band-edge emission quenching. Their findings highlighted the critical role of carrier lifetimes (∼µs) in making TDs visible in PL imaging based on the PL-dark mode.

However, this method was limited by both imaging contrast (∼0.4) and spatial resolution (∼16 µm) for the TSDs. Additionally, the PL-quenching method can differentiate dislocation types only for treated SiC epilayers with low defect density and low impurity concentrations, but it cannot identify whether TDs are killing or benign. The same group also attempted to identify the TDs in n^+^-SiC substrates using NIR-PL imaging under UV excitation.^30^ While this approach revealed TDs as dark spots in the NIR-PL images, it again suffered from low contrast, limited spatial resolution, and a lack of selectivity for TDs with deep states.

Commercially available surface inspection tools, such as KLA Candela and Lasertec SICA, which integrate optical inspection techniques with PL channels, are widely used in industry for nondestructive inspection of extended defects in SiC epilayers, such as stacking faults and surface topographic defects.^42,43^ However, these surface-sensitive tools are ineffective for detecting TDs due to the weak or nonexistent optically detectable surface features and their nanoscale physical size of TDs.^24^ Consequently, significant discrepancies have been reported even for SiC wafers with similar quality from the same vendors. Furthermore, abundant impurities and subsurface damage can further degrade the performance of these tools by disabling PL channels (PL-dark modes) in SiC bulk substrates.^31,44^

Despite widespread adoption in both academia and industry, PL-based techniques still face several critical limitations and challenges in the inspection of electrically active TDs. First, all TD types, as well as all dislocations within the same type, can serve as nonradiative recombination centers, universally quenching the band-edge emissions and impeding the selective detection of device-relevant TDs. Second, imaging contrast and spatial resolution are constrained by carrier diffusion lengths, limiting these techniques only to laboriously treated SiC epilayers with long carrier lifetimes, while they fail to work for highly defective n^+^-SiC substrates. Lastly, the band-edge PL quenching method provides no electronic information about the nature of the defects.

To provide a comprehensive comparison of PL-based techniques, a comparison table focusing on image quality, technique capabilities, and applicability is included (Fig. S5 for more details). This table compares confocal mapping of band-edge emission, PL imaging with a camera, two-photon excitation of band-edge emission, and commercially available inspection tools with our developed technique. Clearly, our technique outperforms existing methods in imaging quality, capability, and applicability.

Recently, conventional 3D laser scanning confocal microscopy with above-bandgap excitation has been employed to study the morphology and defect emissions of the DEPs (rather than the dislocation lines).^34,35^ It is important to note that these studies primarily focus on the interaction between band-edge emissions and newly created external defects at the etch pits, rather than the inherent deep states of electrically active TDs. In contrast, while we also delineated DEPs to locate the positions of TDs, our primary focus is on the specific emissions originating from the inherent deep states of the dislocation lines. In addition, this partially etched TD configuration—comprising both DEPs and dislocation lines—provides an opportunity to further investigate the correlation between the TD structures (revealed by the DEPs) and the optical features of the dislocation lines.

### 3.2. Implication between optically and electrically active DS-STDs

Despite the high density of TDs in SiC, only a small subset is electrically active during device operation by acting as leakage current paths or charge trapping centers.^16^ P. Fiorenza *et al.* examined the roles of TDs in breakdown behavior using nanoscale structural and electrical techniques on 4H-SiC MOSFET devices under high-temperature reverse bias stress.^14^ They found that the TDs with enhanced conductivity always existed in failed devices, confirming the critical role of certain TDs in breakdown behavior. Recently, the effects of TDs on the breakdown behavior of SiC Schottky barrier diodes have also been comprehensively investigated.^18^ It was found that 88% of the breakdown points are associated with the TSDs in the substrate, also highlighting their critical role in device operation.

Such STDs with deep states are electrically active and capable of bridging terminals, thereby forming leakage current paths through continuous deep states. Similarly, these DS-STDs are also optically active and can be selectively excited *via* the same deep states, producing DS-STD-specific emissions that enable nondestructive defect inspection. This concept is analogous to the light-driven deep-level transient spectroscopy technique, which uses light pulses to excite deep-level defects.

## 4. Conclusion

We developed a confocal subsurface defect-PL spectro-microscopy technique, enabling the selective detection of electrically active DS-STDs in n^+^-SiC substrates. The DS-STD-specific emissions allow for obtaining 2D subsurface PL images with ultrahigh contrast and diffraction-limited spatial resolution. Furthermore, depth-corrected 3D images of the complete DS-STDs were reconstructed from 2D-stack defect-PL images, facilitating detailed studies of the correlation between TD morphologies and their optical features. We also analyzed the defect-PL spectra to uncover the electronic nature of deep states. This nondestructive method enables the reliable detection and identification of device-relevant TDs, advancing in-line defect inspection.

## 5. Experiments

### 5.1. Laser scanning confocal dual-mode mapping and PL spectroscopy

Our technique is based on a dual-mode laser scanning confocal microscope utilizing a 375 nm laser. The optical system integrates two single-photon avalanche photodiodes to perform simultaneous laser backscattering mapping (using a 380 nm band-pass filter) and defect-PL mapping (using a 500 nm long-pass filter). The microscope is equipped with an oil-immersion objective of high numerical aperture (N.A. = 1.4), enabling high spatial resolution and efficient photon collection. A 3D piezo controller attached to an objective ensures precise, automatic control of the laser spot positions. For acquiring the PL spectra, an optical fiber was used to channel the emitted photons to a spectrometer equipped with a photomultiplier tube and a monochromator.

### 5.2. 2D-stack mapping with depth correction for 3D image reconstruction

Due to the large refractive index mismatch between the samples and the surrounding medium, spherical aberration arises, resulting in depth profile compression and imaging distortion. To achieve distortion-free 3D imaging, 2D stack mapping with 150 nm increments was performed using a dual-mode laser-scanning confocal microscope. Subsequently, a series of 2D images were acquired and corrected based on a ray tracing model. These depth-corrected 2D images were then utilized to reconstruct distortion-free 3D images using ImageJ. The recovered 3D images provide comprehensive multi-angle views through lateral and cross-sectional slicing. To validate the depth correction, a wafer with the epitaxial layers of well-defined 10 µm thickness was used as a reference.

## Conflicts of interest

There are no conflicts to declare.

## Supplementary Material

NA-OLF-D5NA00970G-s001

## Data Availability

The authors confirm that the data supporting the findings of this study are available within the article and its supplementary information (SI). Supplementary information is available. See DOI: https://doi.org/10.1039/d5na00970g.
