# Supplementary material for: Nondestructive detection and identification of electrically active threading dislocations in n+-SiC substrates
Source: Nanoscale Adv. 2025 Dec 3;8(1):331–9. doi: 10.1039/d5na00970g (PMC12687909; doi:10.1039/d5na00970g)
Supplement: NA-008-D5NA00970G-s001 [file NA-008-D5NA00970G-s001.pdf]

Supplementary Information (SI) for Nanoscale Advances.
This journal is © The Royal Society of Chemistry 2025

Supporting Information

Nondestructive Detection and Identification of Electrically Active Threading Dislocations in n+-SiC Substrates

Irwan Saleh Kurniawan[#1,2], Russel Cruz Sevilla[#1,2], Hsiu-Ming Hsu[#1,2], Ruth Jeane Soebroto[1,2], Chii-Bin Wu[1,2], Ji-Lin Shen[1,2], Hsiu-Ying Huang[*1,2], Wen-Chung Li[*1,2,3], Chi-Tsu Yuan[*1,2]
[1]Department of Physics, Chung Yuan Christian University, Taoyuan, Taiwan
[2]Research Center for Semiconductor Materials and Advanced Optics, Chung Yuan Christian University, Taoyuan, Taiwan
[3]LEAP Semiconductor Corporation, Taoyuan, Taiwan

Fig. S1 Defect selective etching in highly n-doped 4H-SiC substrates

The n^+-doped 4H-SiC substrates, off-cut at 4° with a doping concentration of ~10^{18} cm^{-3}, were supplied by LEAP Semiconductor Corporation. Defect-selective etching (DSE) was performed using molten KOH at 490°C for 9 min to reveal the surface features of various dislocation types, including screw-type dislocations (STDs), threading edge dislocations (TEDs), and basal plane dislocations (BPDs), as shown below**.** After etching, the samples were thoroughly rinsed with deionized water and examined using optical microscopy to identify and categorize different etch pit morphologies. These etched samples allowed us to precisely locate specific TDs of interest for further analysis using our confocal subsurface defect-PL spectro-microscopy technique. Additionally, this partially etched dislocation configuration—which includes both dislocation etch pits and dislocation lines—can serves as a suitable model system for an in-depth study of the correlation between TD structures and their optical characteristics**.**

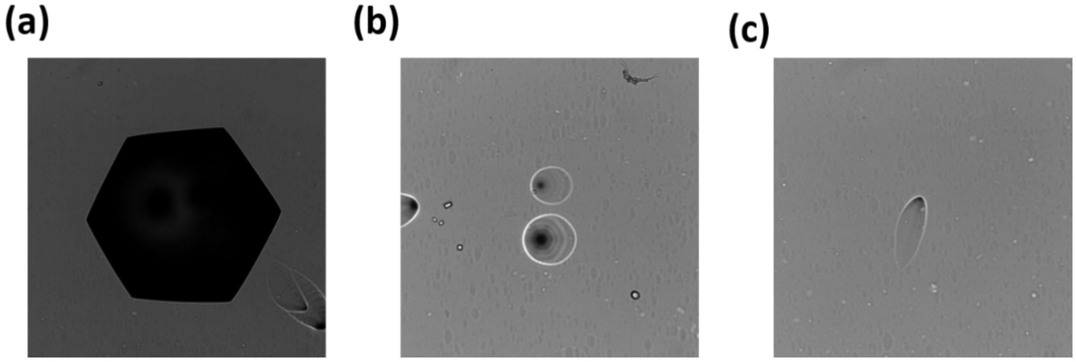

Fig. S2 Statistical analysis of bright electrically active DS-STDs

To validate our findings and estimate the prevalence of electrically active DS-STDs, we analyzed over 300 STDs using the subsurface defect-PL mapping technique described in the main text. Among these, approximately 20 bright DS-STDs were identified, all exhibiting consistent spatial resolution and imaging contrast. Here, we present two additional imaging sets comparing bright DS-STDs and dark STDs, including surface laser backscattering images of etch pits, cross-sectional defect-PL images, and 2D subsurface defect-PL images. Statistical analysis revealed that approximately 7% of STDs in n^+-SiC substrates exhibited bright emission spots originating from DS-STD-specific emissions.

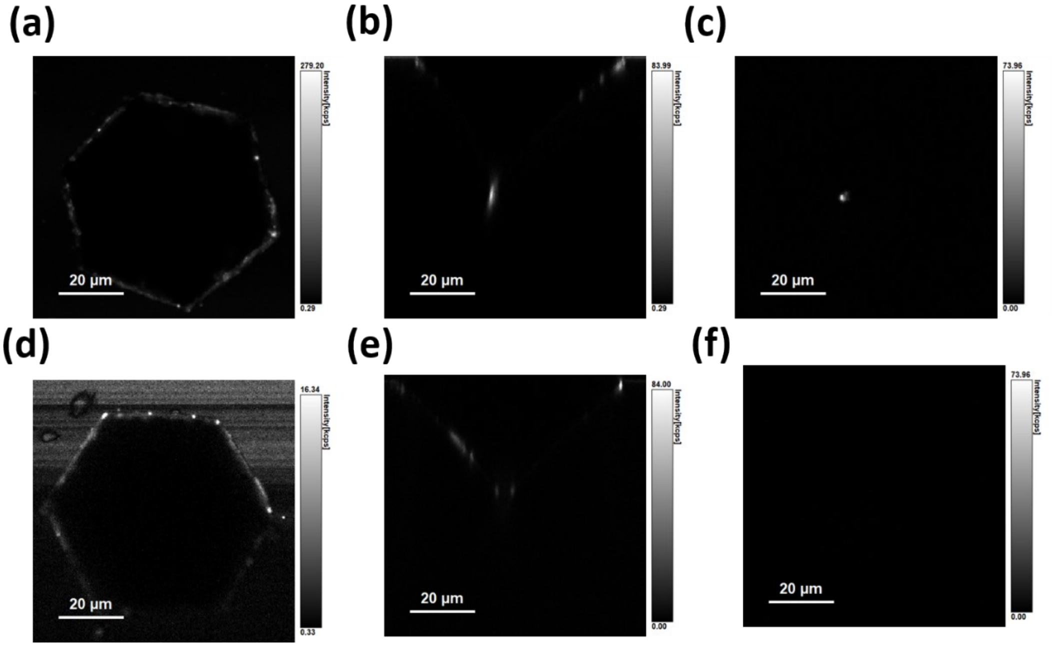

Fig. S3 3D image reconstruction from depth-corrected 2D-stack images

It should be noted that spherical aberration, caused by the refractive index mismatch between the samples and surrounding medium, leads to distortion in cross-sectional image. In our case, the true focal depth of the laser spots extends deeper than the mechanical movement of the microscope objective, resulting in compressed images along the axial direction. Consequently, it is necessary to correct the axial positions of laser focal depths to ensure the construction of accurate 3D imaging.

Diel et al. provided a detailed guideline for axial distortion correction based on ray optics principles, as described in Equation 1:

$$\frac{d'}{d} = \frac{1}{100}\sum_{k=1}^{100}\frac{\tan(\sin^{-1}\frac{kNA}{100n_1})}{\tan(\sin^{-1}\frac{kNA}{100n_2})} \quad (1)$$

Where d'/d is the correction factor related to the actual focal length (d') and expected focal length (d), NA is the numerical aperture of the objective (NA=1.4), and n_1 and n_2 are the refractive index of the immersion medium and the sample (1.52 and 2.62), respectively[1]. The expected focal length refers to the mechanical movement of the microscope objective, which is set according to the Nyquist sampling criterion with consideration of axial spatial resolution. After performing 2D-stack mapping, the actual focal lengths can be calculated using equation 1, and then used to reconstruct distortion-free 3D images by using ImageJ.

To meet the Nyquist sampling criterion, we first determined the depth resolution of our confocal microscope using 375 nm laser excitation and an oil-immersion objective with NA=1.4, based on the equation, $\Delta d = \frac{2n\lambda}{NA^2}$. The depth increment was then set according to the Nyquist condition ($\Delta z < \frac{\Delta d}{2}$). To validate our depth correction, we performed our methods on a SiC epitaxial wafer with a fixed thickness of ~10 μm, and have proved

our calibration.

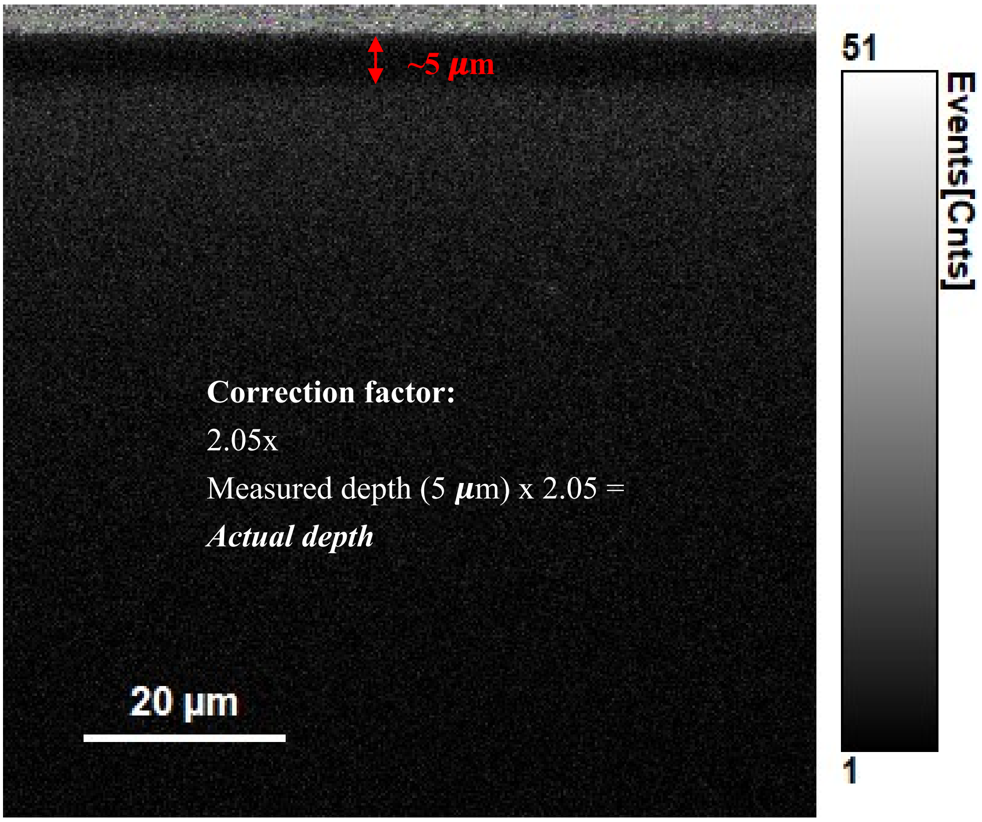

Fig. S4 Gaussian fitting of defect-related PL

In n-type semiconductors, defect-related PL typically arises from two main recombination processes: (1) free-electron to acceptor (e–A^0) transitions, and (2) DAP recombination. To further analyze the origin of our defect-PL spectra, we fitted the spectra using two Gaussian components, as shown below. The two emission peaks, located at 2.5 eV and 2.1 eV, can be attributed to the free-electron–to–bound-hole transition and the DAP recombination, respectively.

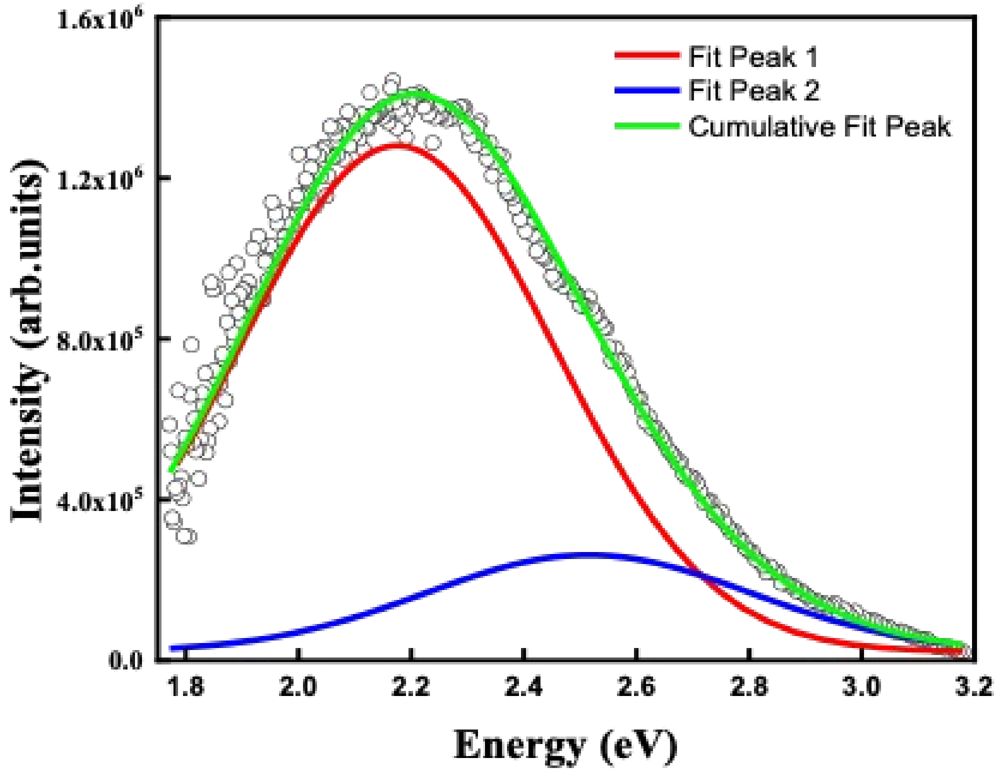

Fig. S5 Comparison of PL-based imaging techniques

To demonstrate the effectiveness of our technique, we compared our experimental data with previously published results and commercially available PL-based techniques, including: (1) confocal micro-PL mapping of band-edge emissions**,** (2) PL imaging with a CCD camera, (3) two-photon-excitation PL imaging of band-edge emissions, (4) commercial defect inspection tools such as Lasertec and KLA. The comparison was conducted in terms of imaging contrast, spatial resolution, detection range, selectivity for electrically active TDs, and applicability to substrates or epilayers. The comparison table and corresponding references are presented below. It is evident that our technique demonstrates superior performance across all criteria, particularly in selectively detecting electrically active TDs in n^+-SiC substrates, a capability not previously achieved.

Work	Technique	Image contrast (contrast ratio)	Spatial resolution (μm)	Selectivity	Applicability	Detection range
1. G. Feng	μ-PL mapping of BEE	0.4	16	No	Epilayers with treatment	UV
2. G. Chung	Commercial inspection tool (Lasertec)	1	19	No	Substrate	NIR
3. C. Kawahara	PL image	0.77	10	No	Epilayers	NIR
4. R. Tanuma	Two-photon excitation of BEE	0.37	12	No	Epilayers	UV
5. Z. Yang	Time-resolved PL mapping	0.16	2.5	No	Epilayers	UV
6. Our work	Confocal subsurface defect-PL mapping, PL-bright mode	~700	1.3~1.6	Yes	Highly doped and undoped substrates	Visible

1. Journal of Applied Physics 110, 033525 (2011)
2. Materials Science Forum 1089, 31 (2023)
3. Japanese Journal of Applied Physics 53, 020304 (2014)
4. Journal of Applied Physics 124, 125703 (2011)
5. Journal of Physical Chemistry Letters 15, 12357 (2024)

Reference

1. G. Feng, J. Suda and T. Kimoto, *Journal of Applied Physics*, 2011, **110**, 033525.
